# Improving the Electroluminescence Properties of New Chrysene Derivatives with High Color Purity for Deep-Blue OLEDs

**DOI:** 10.3390/ma17081887

**Published:** 2024-04-19

**Authors:** Sunwoo Park, Changyu Lee, Hayoon Lee, Kiho Lee, Hyukmin Kwon, Sangwook Park, Jongwook Park

**Affiliations:** Integrated Engineering, Department of Chemical Engineering, Kyung Hee University, Yongin-si 17104, Republic of Korea; mdrafix@khu.ac.kr (S.P.); cksrb712@khu.ac.kr (C.L.); kssarang1@khu.ac.kr (H.L.); kiholee@khu.ac.kr (K.L.); hm531@khu.ac.kr (H.K.); pswook@khu.ac.kr (S.P.)

**Keywords:** asymmetric structure, terphenyl, triphenyl amine, tetraphenylethylene

## Abstract

Two blue-emitting materials, 4-(12-([1,1′:3′,1″-terphenyl]-5′-yl)chrysen-6-yl)-N,N-diphenylaniline (TPA-C-TP) and 6-([1,1′:3′,1″-terphenyl]-5′-yl)-12-(4-(1,2,2-triphenylvinyl)phenyl)chrysene (TPE-C-TP), were prepared with the composition of a chrysene core moiety and terphenyl (TP), triphenyl amine (TPA), and tetraphenylethylene (TPE) moieties as side groups. The maximum photoluminescence (PL) emission wavelengths of TPA-C-TP and TPE-C-TP were 435 and 369 nm in the solution state and 444 and 471 nm in the film state. TPA-C-TP effectively prevented intermolecular packing through the introduction of TPA, a bulky aromatic amine group, and it showed an excellent photoluminescence quantum yield (PLQY) of 86% in the film state. TPE-C-TP exhibited aggregation-induced emission; the PLQY increased dramatically from 0.1% to 78% from the solution state to the film state. The two synthesized materials had excellent thermal stability, with a high decomposition temperature exceeding 460 °C. The two compounds were used as emitting layers in a non-doped device. The TPA-C-TP device achieved excellent electroluminescence (EL) performance, with Commission Internationale de L′Eclairage co-ordinates of (0.15, 0.07) and an external quantum efficiency of 4.13%, corresponding to an EL peak wavelength of 439 nm.

## 1. Introduction

Research on blue-light-emitting materials has attracted much attention since the development of organic light-emitting diodes (OLEDs) [1,2]. Furthermore, research on blue inorganic LEDs utilizing inorganic materials such as III-nitrides (AlN, InN, and GaN) has also been reported. Among these, GaN-based LEDs, in particular, have garnered significant attention due to their robustness and capability to achieve high brightness and long lifetimes [3,4]. Stable and highly efficient deep-blue-light-emitting materials with Commission Internationale de L′Eclairage (CIE) co-ordinates of (0.14, 0.08), as indicated by the National Television System Committee (NTSC), are necessary for new device development. However, the wide band gap of the blue emitter is a significant problem, as it requires a high driving voltage, which can cause deterioration and reduce efficiency over time. For this reason, despite the many studies conducted on deep-blue-emitting materials, only a few studies have achieved both high efficiency and pure deep-blue emission [5,6,7,8,9].

High-efficiency fluorescent blue emitters can be attained using a core-side molecular design. In this approach, a central core chromophore is selected that determines the luminous efficiency and band gap of the material; side groups are then introduced into the core group to control the molecular arrangement, polarity, and emission wavelength [10]. Considerable efforts have focused on the use of anthracene and pyrene chromophores as cores to achieve blue emission; however, these molecules show real blue or sky-blue emission when a side group is introduced due to the intrinsic conjugation length [11,12,13,14,15,16,17,18]. Chrysene, on the other hand, has a short conjugation length and exhibits a wide band gap, making it suitable for use as a deep-blue luminous core. However, the problem of intermolecular stacking and a red shift in the emission wavelength caused by the planar structure of core molecules should be prevented by introducing side groups. Depending on the type and position, side groups can improve the efficiency of the entire molecule by solving these problems. Additionally, specific side groups can induce characteristics such as aggregation-induced emission (AIE), which is characterized by strong luminescence during molecular aggregation and a hybridized local and charge-transfer (HLCT) excited state, theoretically exhibiting a characteristic of maximum exciton utilization efficiency (EUE) of 100% due to the reverse intersystem crossing process (RISC) occurring at high-lying energy levels [19].

In this study, we synthesized two high-efficiency deep-blue fluorescent emitters: 4-(12-([1,1′:3′,1″-terphenyl]-5′-yl)chrysen-6-yl)-N,N-diphenylaniline (TPA-C-TPA) and 6-([1,1′:3′,1″-terphenyl]-5′-yl)-12-(4-(1,2,2-triphenylvinyl)phenyl)chrysene (TPE-C-DPA). These emitters were designed asymmetrically by introducing terphenyl (TP), triphenyl amine (TPA), or tetraphenylethylene (TPE) on both sides of the chrysene core. TP was used instead of triphenylbenzene to suppress excimer formation through an optimized steric effect. TPA maintains the emission wavelength of the core chromophore and enhances hole injection characteristics through electron-donating properties. These bulky side groups are often used to increase electroluminescence (EL) efficiency by preventing intermolecular packing in the film state and forming an amorphous thin film. The asymmetric structure with TPA was substituted only on one side, showing weak bipolar characteristics that could lead to HLCT characteristics. TPE induces an AIE effect to improve luminous efficiency based on a high photoluminescence quantum yield (PLQY) in the film state. It has been reported that when TPE is symmetrically substituted on both sides of the core, such as anthracene and pyrene, strong molecular aggregation occurs, resulting in a decrease in luminous efficiency and a red shift in the luminous wavelength [20,21,22,23,24]. For this reason, TPE was substituted asymmetrically [25,26,27]. Notably, there is no report on the application of the AIE effect on chrysene to date.

## 2. Materials and Methods

### 2.1. Materials and Instrumentation

All reagents and solvents were purchased from Sigma-Aldrich (St. Louis, MO, USA), TCI, and Alpha Aesar corporation and used without further purification. The ^1^H-NMR spectra were recorded on Bruker Advance 300 spectrometers (Billerica, MA, USA). The optical absorption spectra were obtained by using an Agilent 8453 ultraviolet–visible (UV-Vis) spectrometer (Santa Clara, CA, USA). A Perkin Elmer luminescence spectrometer LS55 (Xenon flash tube) was used for photoluminescence (PL) spectroscopy (Waltham, MA, USA). Absolute photoluminescence quantum yield (PLQY) values were obtained by using Quantaurus-QY (Hamamatsu, Japan). The DFT-based calculations on TPA-C-TP and TPE-C-TP were performed with the B3LYP functional and the 6-31G(d) basis set using the NWChem 6.8.1 program package [28]. The glass transition temperatures (Tg) and melting temperatures (Tm) of the compounds were determined using a Differential Scanning Calorimetry (DSC) Discovery DSC25 (TA Instruments, New Castle, DE, USA) under a nitrogen atmosphere, employing heating and cooling rates of 10 °C/min, with the compounds heated to 350 °C. Thermal gravimetric analysis (TGA) was conducted using a TGA4000 (Perkin Elmer), with the samples heated to 700 °C at a rate of 10 °C/min. The HOMO energy levels were assessed using ultraviolet photoelectron yield spectroscopy (Riken Keiki AC-2, Tokyo, Japan), while the LUMO energy levels were calculated from the HOMO energy levels and the band gaps. The organic layers for EL devices were deposited under a vacuum of 10^−6^ torr, with a deposition rate of 1 Å/s, resulting in a deposition area of 4 mm^2^. The geometry of one device is 2.5 cm × 2.5 cm, with four emission spots, each having an emitting area of 4 mm^2^. The continuous deposition of LiF and aluminum layers was carried out under the same vacuum conditions. The current-voltage-luminance (I-V-L) characteristics of the fabricated EL devices were measured using a Keithley 2400 electrometer (Cleveland, OH, USA). Luminance was measured with a Minolta CS-1000A (Konica Minolta, Tokyo, Japan).

### 2.2. Synthesis

**Scheme 1:**
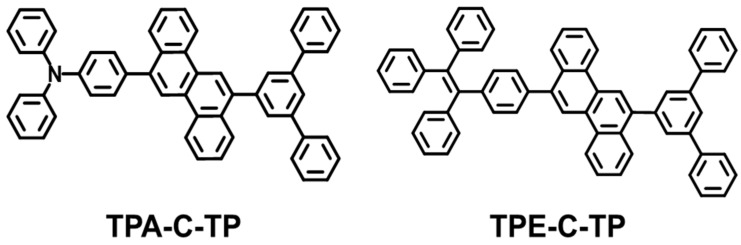
Molecular structures of the newly synthesized blue emitters: TPA-C-TP and TPE-C-TP.

**Scheme 2:**
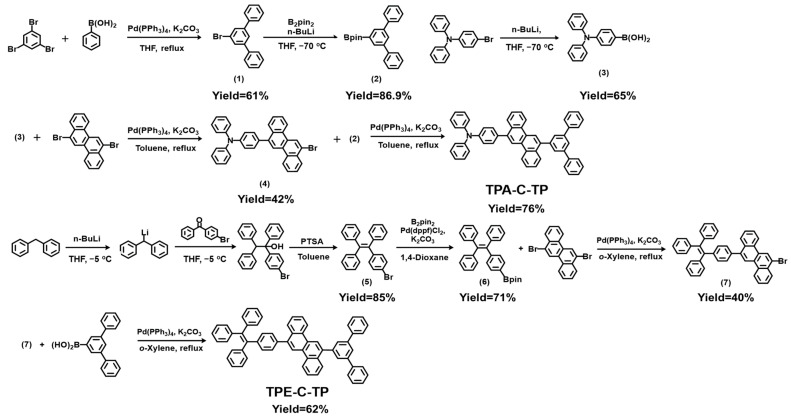
Synthetic routes of the synthesized materials.

New blue-emitting materials, 4-(12-([1,1′:3′,1″-terphenyl]-5′-yl)chrysen-6-yl)-N,N-diphenylaniline (TPA-C-TP) and 6-([1,1′:3′,1″-terphenyl]-5′-yl)-12-(4-(1,2,2-triphenylvinyl)phenyl)chrysene (TPE-C-TP), were described in Scheme 1 and Scheme 2 and were reported in a previous study [29,30,31]. The relevant NMR data are presented in Figures S1–S3.

#### 2.2.1. Synthesis of 4-(12-([1,1′:3′,1″-terphenyl]-5′-yl)chrysen-6-yl)-N,N-diphenylaniline—TPA-C-TP

In a 100 mL round-bottom flask, we added 0.35 g (1.4 mmol) of 6-([1,1′:3′,1″-terphenyl]-5′-yl)-12-bromochrysene (4) and 0.04 g (0.07 mmol) of tetrakis(triphenylphosphine)palladium(0) to 40 mL of anhydrous toluene solution. Then, we heated the reaction mixture to 110 °C for 4 h under nitrogen. Following this, we utilized chloroform and water for extraction. After drying the organic layer with anhydrous MgSO_4_ and filtering it, we evaporated the solvent. Finally, we purified the product by silica gel column chromatography using the CHCl3:hexane (1:4) eluent, yielding a white solid (yield: 76%). ^1^H NMR (400 MHz, THF-d_8_): δ(ppm) = 9.00 (d, J = 8.7 Hz, 2H), 8.88 (s, 1H), 8.78 (s, 1H), 8.13 (d, J = 8.3 Hz, 2H), 8.03 (t, J = 1.7 Hz, 1H), 7.90 (d, J = 1.7 Hz, 2H), 7.82–7.79 (m, 4H), 7.67 (dddd, J = 12.5, 8.3, 6.8, 1.4 Hz, 2H), 7.59–7.54 (m, 4H), 7.44 (dd, J = 8.4, 7.0 Hz, 4H), 7.35–7.31 (m, 2H), 7.30–7.26 (m, 4H), 7.25–7.22 (m, 2H), 7.19–7.16 (m, 4H), 7.02 (tt, J = 7.2, 1.3 Hz, 2H).

#### 2.2.2. Synthesis of 6-bromo-12-(4-(1,2,2-triphenylvinyl)phenyl)chrysene—(**7**)

In a 500 mL round-bottom flask, we added 2 g (5.1 mmol) of 6,12-dibromochrysene, 2.85 g (6.2 mmol) of 4,4,5,5-tetramethyl-2-(4-(1,2,2-triphenylvinyl)phenyl)-1,3,2-dioxaborolane (6), 2.5 g (18 mmol) of potassium carbonate, and 0.3 g (0.26 mmol) of tetrakis(triphenylphosphine)palladium(0). This mixture was dissolved in 200 mL of anhydrous o-xylene and 40 mL of distilled water and then stirred. The reaction mixture was heated to 110 °C for 20 h under nitrogen. Upon completion of the reaction, it was extracted with chloroform and distilled water, and the organic layer was dried with anhydrous MgSO_4_. The product was subsequently purified by silica gel column chromatography using the CHCl3:hexane (1:9) eluent, yielding a white solid (yield: 40%). ^1^H NMR (400 MHz, DMSO-d_6_): δ(ppm) = 9.27 (s, 1H), 9.06–8.99 (m, 2H), 8.67 (s, 1H), 8.33 (dd, J = 7.6, 1.9 Hz, 1H), 7.84–7.73 (m, 4H), 7.68–7.62 (m, 1H), 7.40–7.35 (m, 2H), 7.24–7.13 (m, 9H), 7.13–7.05 (m, 6H), 7.03–6.99 (m, 2H).

#### 2.2.3. Synthesis of 6-([1,1′:3′,1″-terphenyl]-5′-yl)-12-(4-(1,2,2-triphenylvinyl)phenyl)chrysene—TPE-C-TP

In a 250 mL round-bottom flask, we added 1.3 g (2 mmol) of compound (7), 0.62 g (2.2 mmol) of [1,1′:3′,1″-terphenyl]-5′-ylboronic acid, 0.91 g (6.5 mmol) of potassium carbonate, and 0.11 g (0.09 mmol) of tetrakis(triphenylphosphine)palladium(0). This mixture was dissolved in 140 mL of anhydrous o-xylene and 30 mL of distilled water and then stirred. The reaction mixture was heated to 110 °C for 20 h under nitrogen. Upon completion of the reaction, it was extracted with dichloromethane and distilled water, and the organic layer was dried with anhydrous MgSO_4_. The product was subsequently purified by silica gel column chromatography using the CHCl_3_:hexane (2:8) eluent, yielding a white solid (yield: 62%). ^1^H NMR (400 MHz, DMSO-d_6_): δ(ppm) = 9.13 (d, J = 8.2 Hz, 1H), 9.04 (d, J = 8.6 Hz, 1H), 8.93 (s, 1H), 8.72 (s, 1H), 8.07 (d, J = 8.2 Hz, 1H), 8.03 (s, 1H), 7.89 (s, 2H), 7.87 (s, 4H), 7.79 (d, J = 9.3 Hz, 1H), 7.75–7.62 (m, 4H), 7.49 (t, J = 7.6 Hz, 4H), 7.43–7.37 (m, 4H), 7.25–7.14 (m, 9H), 7.13–7.07 (m, 6H), 7.02 (s, 2H).

## 3. Results and Discussion

### 3.1. Molecular Design, Synthesis, and Characterization of Organic Blue Emitters

Scheme 1 and Scheme 2 show the chemical structure and synthesis method used to make the material, using the well-known and generally simple Suzuki coupling and McMurry coupling methods and borylation reactions; more detailed information on these processes is provided in the Section 2 and Scheme 2. The synthesized compounds were characterized using nuclear magnetic resonance spectroscopy, as shown in Figures S1–S3 (see Supplementary Materials). TPA-C-TP and TPE-C-TP were synthesized using chrysene as a core suitable for deep-blue emission, and both contained a bulky TP moiety. Here, TPA with electron-donating ability and TPE with AIE characteristics were substituted to maintain deep-blue emission with an asymmetric structure. In the case of compounds with asymmetric structures, intermolecular packing is effectively prevented in the film state, resulting in amorphous films and improved device stability [32,33,34]. The optical and electrical properties of synthesized blue chrysene derivatives were compared and analyzed.

### 3.2. Photophysical Properties

**Figure 1:**
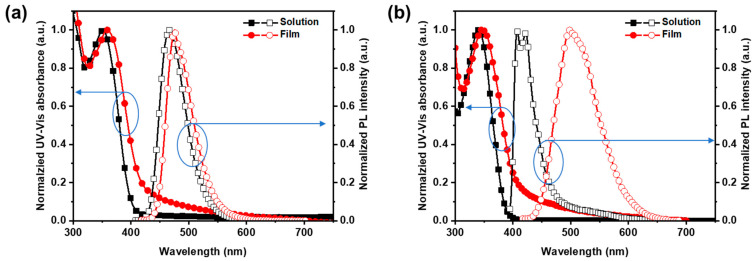
Ultraviolet–visible absorption spectra and photoluminescence (PL) spectra of the synthesized materials: (**a**) TPA-C-TP and (**b**) TPE-C-TP in toluene solution (1.0 × 10^−5^ M) and (**b**) in the evaporated thin-film state (thickness: 50 nm).

Ultraviolet–visible absorption and photoluminescence (PL) measurements were conducted to evaluate the photophysical properties of the synthesized chrysene derivatives, TPA-C-TP and TPE-C-TP; the spectra of these compounds in the solution and film states and their characteristics are shown in Figure 1 and are listed in Table 1. 

The maximum absorption wavelengths of TPA-C-TP and TPE-C-TP in the solution state were 352 and 340 nm, respectively; this is a difference of 27 and 15 nm when compared to the intrinsic absorption wavelength of 325 nm of the chrysene chromophore, as the intramolecular conjugation length was increased slightly by the introduction of a side group. The absorption wavelength of TPA-C-TP was red-shifted by 12 nm compared to TPE-C-TP; in this case, the band gap was reduced due to the electron-donating effect of the TPA side group. In the film state, the maximum absorption wavelength of TPA-C-TP and TPE-C-TP were 359 and 348 nm, respectively, and showed similar tendencies to the solution state. 

**Figure 2:**
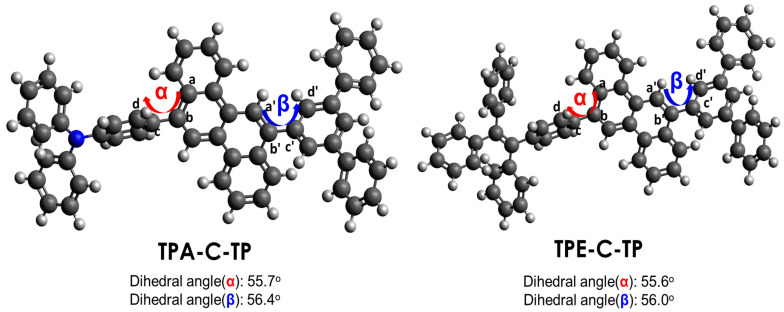
Optimized structures and dihedral angles of the chrysene derivatives: TPA-C-TP and TPE-C-TP calculated at the B3LYP/6-31G(d) theory level (large black ball: carbon; small white ball: hydrogen; large blue ball: nitrogen).

The maximum photoluminescence emission wavelength (PL_max_) of TPA-C-TP and TPE-C-TP in the solution state were 435 and 369 nm, respectively, and 444 and 471 nm in the film state. All of the newly synthesized materials showed emission in the deep-blue region in the solution state, whereas only TPA-C-TP realized emission in the deep-blue region in the film state. In the case of TPA-C-TP, PL_max_ was red-shifted 10 nm in the film state compared to the solution state, and the full width at half maximum (FWHM) values were similar at 56 and 58 nm, respectively. PL_max_ is commonly red-shifted in the film state compared with the solution state, and the FHWM becomes broad due to the π-π interactions caused by the narrowing of the intermolecular distance. However, the intermolecular packing was effectively prevented by the substituted side groups of TPA and TP; thus, PL_max_ was slightly shifted, and the FWHM remained nearly the same. As determined by the molecular calculations, the dihedral angle (a-b-c-d, α position) between the chrysene and TPA of TPA-C-TP was 55.7°, and the dihedral angle (a′-b′-c′-d′, β position) between chrysene and TP was 56.4° (Figure 2). Here, intermolecular packing can be effectively prevented in the film state due to steric hindrance between the core and the side groups. 

PL_max_ in the film state of TPE-C-TP showed a red shift of 102 nm compared to the value in the solution state, and the FWHM increased by 49 nm. TPE is known to be a bulky side group. The dihedral angle (a-b-c-d, α position) between the chrysene and TPE of TPE-C-TP was 55.7° and that (a′-b′-c′-d′, β position) of chrysene and TP was 56.4°, similar to the dihedral angle of TPA-C-TP. Thus, the interaction between the molecules can be suppressed. However, due to the limited movement from the double bond of TPE, the molecules in the film state tend to aggregate, resulting in a large Stokes shift and a broad FWHM. This is a common feature of materials that exhibit the AIE phenomenon. In contrast, in the solution state, the PL_max_ of TPE-C-TP was blue-shifted by 66 nm compared to that of TPA-C-TP (red-shifted, with high electron-donating ability). TPE-C-TP prevented intramolecular conjugation from lengthening due to the molecular distortion caused by the TPE group, thereby showing emission at short wavelengths.

In the solution state, the PLQY values of TPA-C-TP and TPE-C-TP were 91% and 0.1%, respectively. In the film state, the PLQY values of TPA-C-TP and TPE-C-TP were 86% and 78%, respectively. In general, as the distance between molecules in the film state decreases, π-π interactions occur between the molecules, leading to aggregation-caused quenching (ACQ), the red-shifting of PL_max_, and a decrease in PLQY. However, in the case of TPA-C-TP, the side groups TPA and TP maintained a sufficient distance between the molecules to effectively prevent ACQ, showing excellent PLQY values in both the solution and film states. In the case of TPE-C-TP, in the solution phase, the non-radiative decay was dominant because TPE strongly induced vibration and rotational motion, showing a low PLQY value. In the film state, the molecular motion that causes non-radiative decay is restricted due to molecular aggregation, which promotes radiative decay and, thus, provides high luminous efficiency with AIE characteristics.

**Figure 3:**
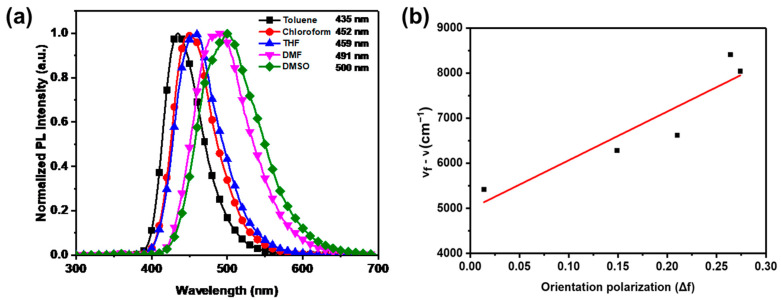
(**a**) PL spectra of TPA-C-TP according to solvents with different dielectric constants; (**b**) fitted linear correlation of the Stokes shift as a function of solvent polarity for TPA-C-TP.

TPA-C-TP displayed weak bipolar characteristics due to TPA having an electron-donating effect. Solvatochromic shifts were investigated in solvents with different polarities, specifically toluene, chloroform, tetrahydrofuran, dimethylformamide, and dimethyl sulfoxide (DMSO), with dielectric constants of 2.38, 4.81, 7.58, 36.7, and 46.68, respectively. Figure 3a shows the PL spectra with the solvatochromic shift for the different solutions. The PL_max_ of TPA-C-TP was red-shifted from 435 nm (toluene) to 500 nm (DMSO) as the solvent polarity increased. In this regard, the changes in Stokes shift with respect to solvent polarity were plotted and can be seen in Figure 3b. It can be observed that as the polarity values of the solvents increase, the Stokes shift values linearly increase. Such HLCT form implies a combination of the locally excited (LE) state and charge transfer (CT) state, resulting in highly efficient radiative characteristics and weakly bound properties [35,36].

**Figure 4:**
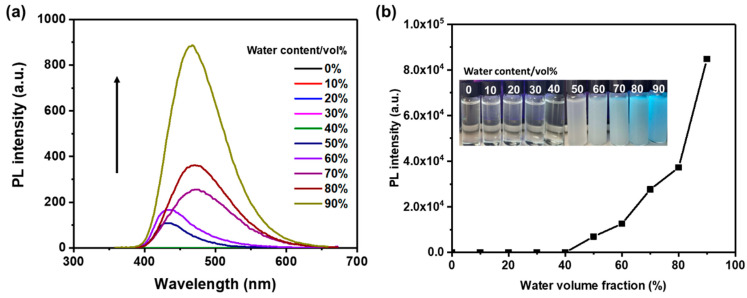
(**a**) PL spectra of TPE-C-TP in H_2_O/THF mixtures (10 × 10^−5^ M) as a function of wavelength and water content (excitation wavelength: 340 nm). (**b**) Plot of I/I_0_ versus the water volume fraction, where I_0_ is the emission intensity in pure THF solution. The inset shows the emission image displays under radiation with 365 nm UV.

The AIE characteristics of TPE-C-TP were confirmed by examining the PL spectra of the diluted solutions in THF-water-solvent mixtures of various volume fractions (Figure 4). The PL intensity continued to increase with water content. When the water content reached 90%, a remarkable rise of about 880% was observed when compared to pure THF. This indicates that, with an increase in non-solvent water content, the molecular motion that caused non-radiative decay was suppressed, and the PL intensity increased, showing AIE.

### 3.3. Electroluminescence Properties

**Figure 5:**
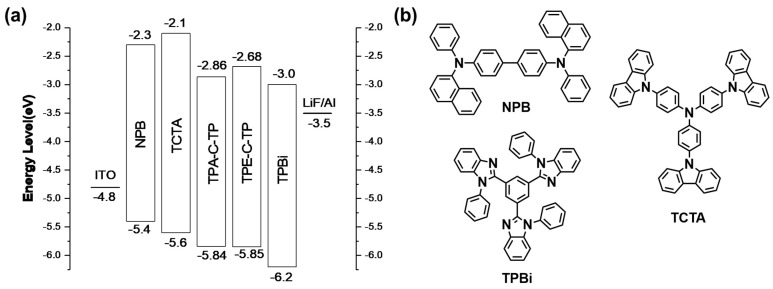
(**a**) Device structure and energy level diagrams of the fabricated OLED devices and (**b**) the chemical structures of organic materials used in the fabricated devices.

**Figure 6:**
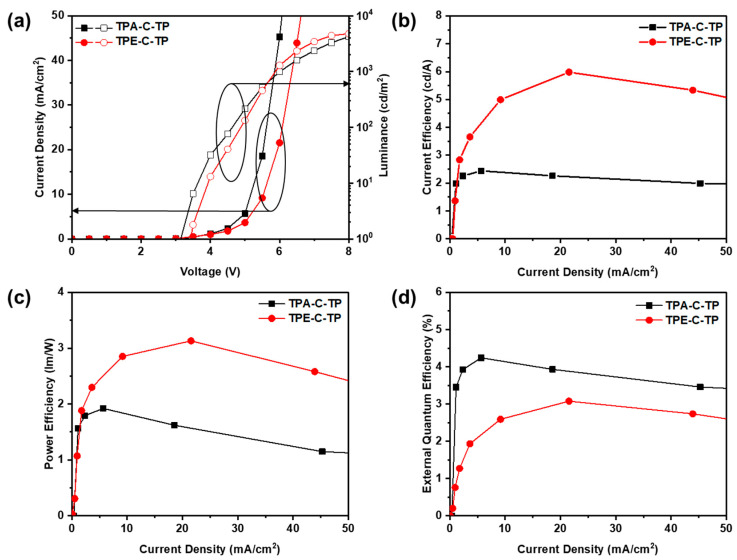
Device performance of TPA-C-TP and TPE-C-TP: (**a**) current density and luminance-voltage; (**b**) current efficiency–current density; (**c**) power efficiency–current density; (**d**) external quantum efficiency–current density curves.

**Figure 7:**
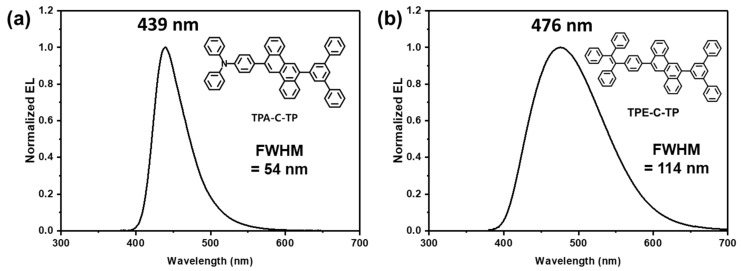
Normalized EL spectra of the synthesized compounds: (**a**) TPA-C-TP and (**b**) TPE-C-TP.

In order to confirm the EL properties of the three tested light-emitting materials, each of them was used as an emitting layer (EML) in an OLED device. In addition, a non-doped device was fabricated to maximize the AIE effect in TPE-C-TP. The OLED device architecture comprised a layer of indium tin oxide (ITO, 150 nm) serving as the anode, succeeded by N,N′-bis(naphthalen-1-yl)-N,N′-bis(phenyl)benzidine (NPB) (40 nm), tris-(4-carbazoyl-9-ylphenyl)amine (TCTA) (15 nm), the custom synthesized emissive materials (30 nm), 1,3,5-tri(1-phenyl-1H-benzo[d]imidazol-2-yl)phenyl (TPBi) (20 nm), LiF (1 nm), and aluminum (Al, 200 nm) acting as the cathode. Figure 5 displays the chemical structures of the materials and band diagrams for the OLED devices. Here, NPB was used as the hole injection layer (HIL), and TCTA was used as the hole transport layer (HTL). TPBi was used as an electron transporting layer (ETL), LiF provided the electron injection layer (EIL), and ITO and Al were used as the anode and cathode, respectively. Figure 6a–d show the determined current density (J)–voltage (V)–luminance (L), current efficiency (CE)-J, power efficiency (PE)-J, and EQE-J curves of the OLED devices. The EL performance data of the devices are summarized in Figure 7 and Table 2 at a current density of 10 mA/cm^2^. TPA-C-TP exhibited deep-blue emission with CIE (x, y) co-ordinates of (0.15, 0.07), a CE of 2.39 cd/A, a PE of 1.82 lm/W, and an EQE of 4.13%. TPE-C-TP exhibited sky-blue emission with CIE co-ordinates of (0.19, 0.28), a CE of 5.05 cd/A, a PE of 2.87 lm/W, and an EQE of 2.62%. TPA-C-TP showed a 1.5-fold higher EQE value compared to TPE-C-TP. TPA-C-TP exhibited not only a high PLQY due to the donating effect of TPA but also a shallower HOMO level than TPE-C-TP; thus, the energy barrier between the HTL and EML layers was small, facilitating electron injection with a low driving voltage. TPE-C-TP showed a higher CE than TPA-C-TP due to the AIE effect, but, at the same time, the EQE efficiency was low due to the low color purity from the broadening of the FHWM. The maximum EL wavelengths of the devices using TPA-C-TP and TPE-C-TP were 439 and 476 nm, respectively, and the FWHM values were 54 and 113 nm, respectively. These values were similar to the PL_max_ values of the materials in the film state. Therefore, among the two synthesized materials, TPA-C-TP showed excellent color purity and superior EL performance, as indicated by the CIE y-value, and is suitable for high-definition televisions that require a CIE y-value of 0.08, as reported by the NTSC.

## 4. Conclusions

Novel chrysene derivatives—TPA-C-TP and TPE-C-TP—were successfully synthesized. In the newly synthesized compounds, chrysene was set as the central core, and TP, TPA, and TPE were introduced as side groups. The PLQY values of TPA-C-TP and TPE-C-TP were 86 and 78% in film, respectively. The high PLQY of TPA-C-TP was attributed to both the steric effect of the side group and the presence of HLCT features. In the case of TPE-C-TP, AIE was observed due to TPE, which improved the PLQY of the film state. When the synthesized material was used as an emitting layer in a non-doped OLED, the EQE values of TPA-C-TP and TPE-C-TP were 4.13% and 2.62%, respectively. Notably, TPA-C-TP exhibited deep-blue emission with CIE co-ordinates of (0.15, 0.07), thus satisfying the NTSC requirement. These results are expected to contribute to the development of new blue-light-emitting technology.

## Figures and Tables

**Table 1:** Photophysical properties of the synthesized materials.

	Solution State ^a^			Film State ^b^			T_g_^d^ (°C)	T_m_^e^ (°C)	T_d_^f^ (°C)	PLQY ^g^ (%) sol	PLQY ^g^ (%) film	HOMO ^h^ (eV)	LUMO ^i^ (eV)	Bandgap (eV)
	UV_max_ (nm)	PL_max_ (nm)	FWHM ^c^ (nm)	UV_max_ (nm)	PL_max_ (nm)	FWHM ^c^ (nm)								
TPA-C-TP	352	435	56	359	444	58	-	256	467	92	86	−5.61	−2.63	2.98
TPE-C-TP	340	369	44	348	471	93	150	297	460	0.1	78	−5.85	−2.68	3.17

^a^ Toluene solution (1.0 × 10^−^^5^ M); ^b^ evaporated film with a thickness of 50 nm on glass; ^c^ full width at half maximum (FWHM) of PL peak; ^d^ glass transition temperature; ^e^ melting temperature; ^f^ 5% weight loss; ^g^ absolute photoluminescence quantum yield; ^h^ HOMO level was measured using ultraviolet photoelectron spectroscopy (Riken-Keiki; AC-2); ^i^ LUMO obtained from HOMO and the optical band gap.

**Table 2:** EL performances of the fabricated OLED devices at 10 mA/cm^2^.

Materials	V_on_ ^a^ (V)	CE ^b^ (cd/A)	PE ^c^ (lm/W)	EQE ^d^ (%)	CIE ^e^ (x, y)	EL_max_ (nm)	FWHM (nm)
TPA-C-TP	5.17	2.39	1.82	4.13	(0.15, 0.07)	439	54
TPE-C-TP	5.55	5.05	2.87	2.62	(0.19, 0.28)	476	114

^a^ Operating voltage at 10 mA/cm^2^; ^b^ luminance efficiency; ^c^ power efficiency; ^d^ external quantum efficiency; ^e^ Commission International de L’Eclairage co-ordinates.

## Data Availability

Data are contained within this article and Supplementary Materials.

## Supplementary Materials

The following Supporting Information can be downloaded at: https://www.mdpi.com/article/10.3390/ma17081887/s1, Figure S1: ^1^H-NMR spectrum of TPA-C-TP; Figure S2: ^1^H-NMR spectrum of compound (7); Figure S3: ^1^H-NMR spectrum of TPE-C-TP; Figure S4: The characteristics of the natural transition orbitals (NTO) for the S1 state of TPA-C-TP, calculated using TDA-DFT B3LYP/6-31G(d) function; Figure S5: Frontier molecular orbital distributions of TPA-C-TP and TPE-C-TP calculated at the B3LYP/6-31G(d) level of theory; Figure S6: (a) Thermogravimetric analysis curves and (b) differential scanning calorimetry curves of TPA-C-TP and TPE-C-TP.; Figure S7: EL characteristic of luminance-current density curves for devices using TPA-C-TP and TPE-C-TP as the emitter. Refs. [37,38] are cited in the Supplementary Materials.
