# Supplementary material for: Improving the Electroluminescence Properties of New Chrysene Derivatives with High Color Purity for Deep-Blue OLEDs
Source: Materials (Basel). 2024 Apr 19;17(8):1887. doi: 10.3390/ma17081887 (PMC11052429; doi:10.3390/ma17081887)
Supplement: Supplementary file 1 [file materials-17-01887-s001.zip › materials-2954072-supplementary.pdf]

Improving the Electroluminescence Properties of New Chrysene Derivatives with High Color Purity for Deep-Blue OLEDs

*Sunwoo Park[1,‡], Changyu Lee[1,‡] Hayoon Lee[1], Kiho Lee[1], Hyukmin Kwon [1], Sangwook Park[1], and Jongwook Park[1]**

[1]Integrated Engineering, Department of Chemical Engineering, Kyung Hee University, Gyeonggi, 17104, Korea

[‡]Sunwoo Park, and Changyu Lee contributed equally to this work as first authors.

**E-mail: jongpark@khu.ac.kr*

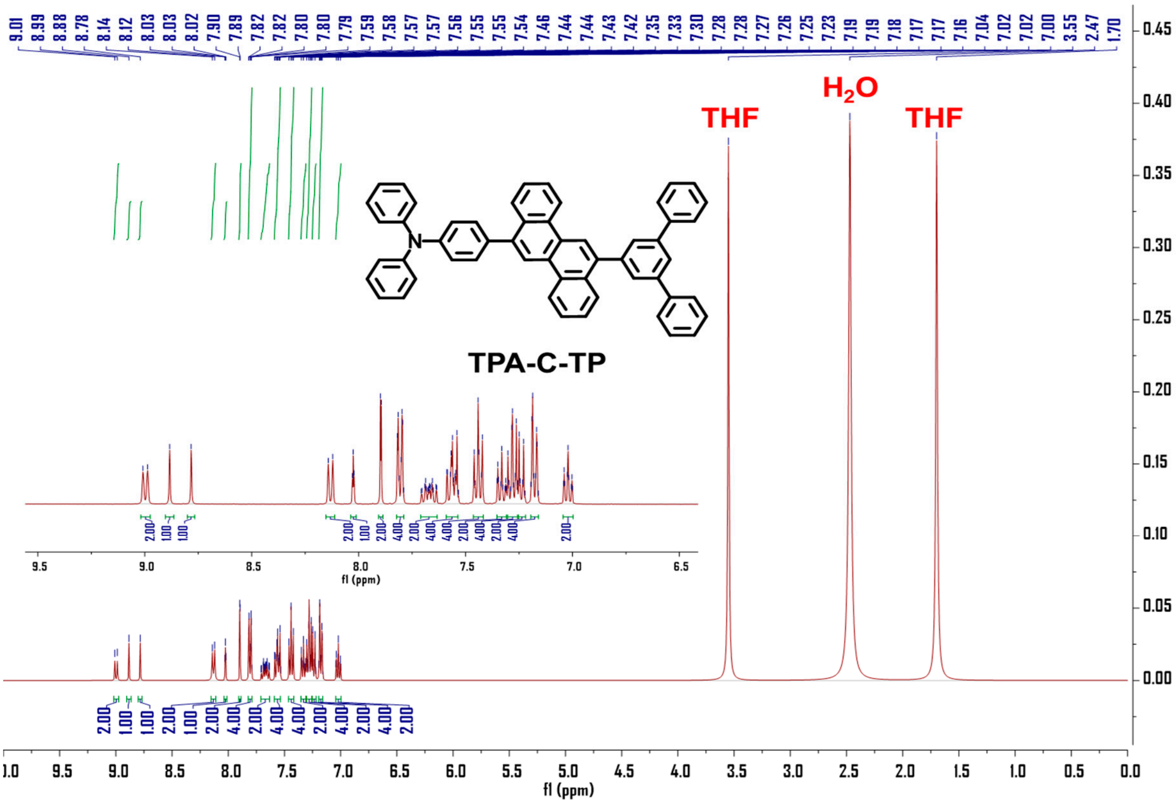

Figure S1. ^{1}H-NMR spectrum of TPA-C-TP.

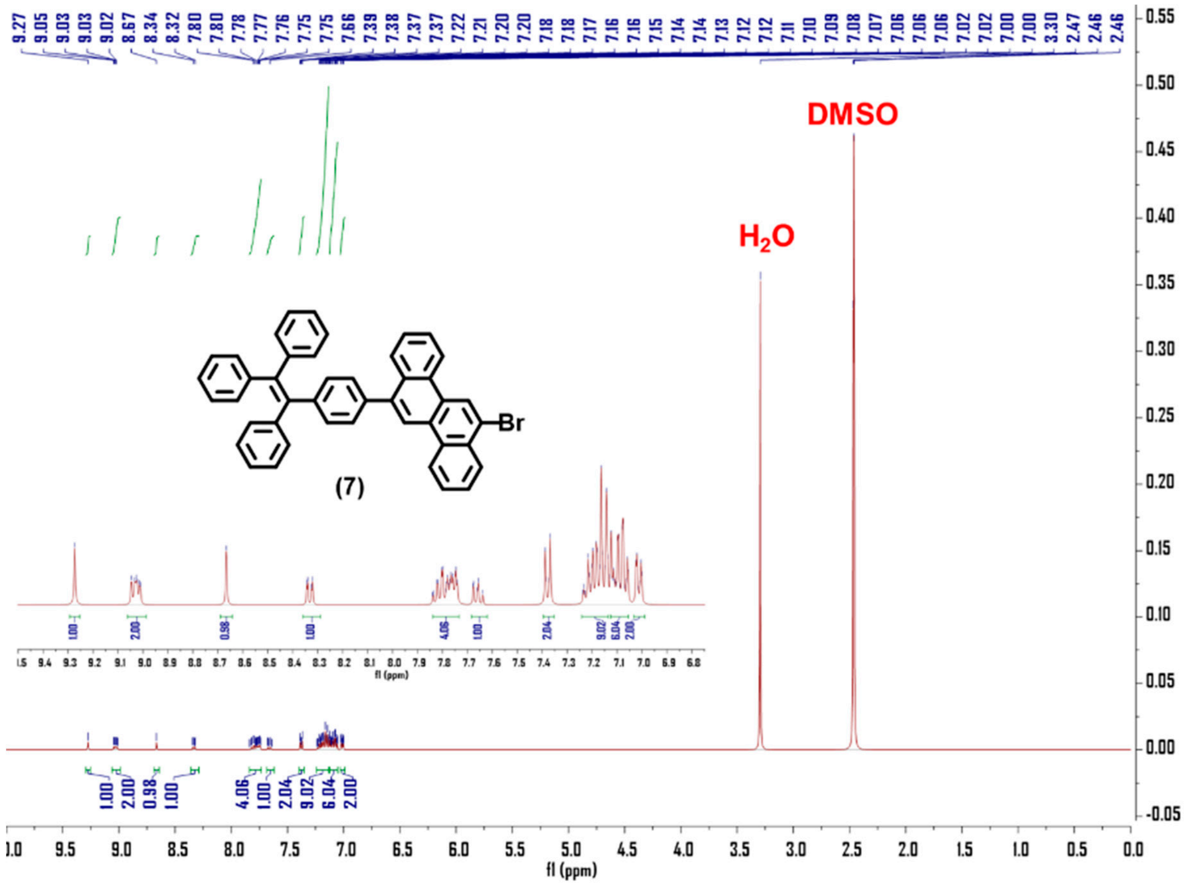

Figure S2. ^{1}H-NMR spectrum of compound (7).

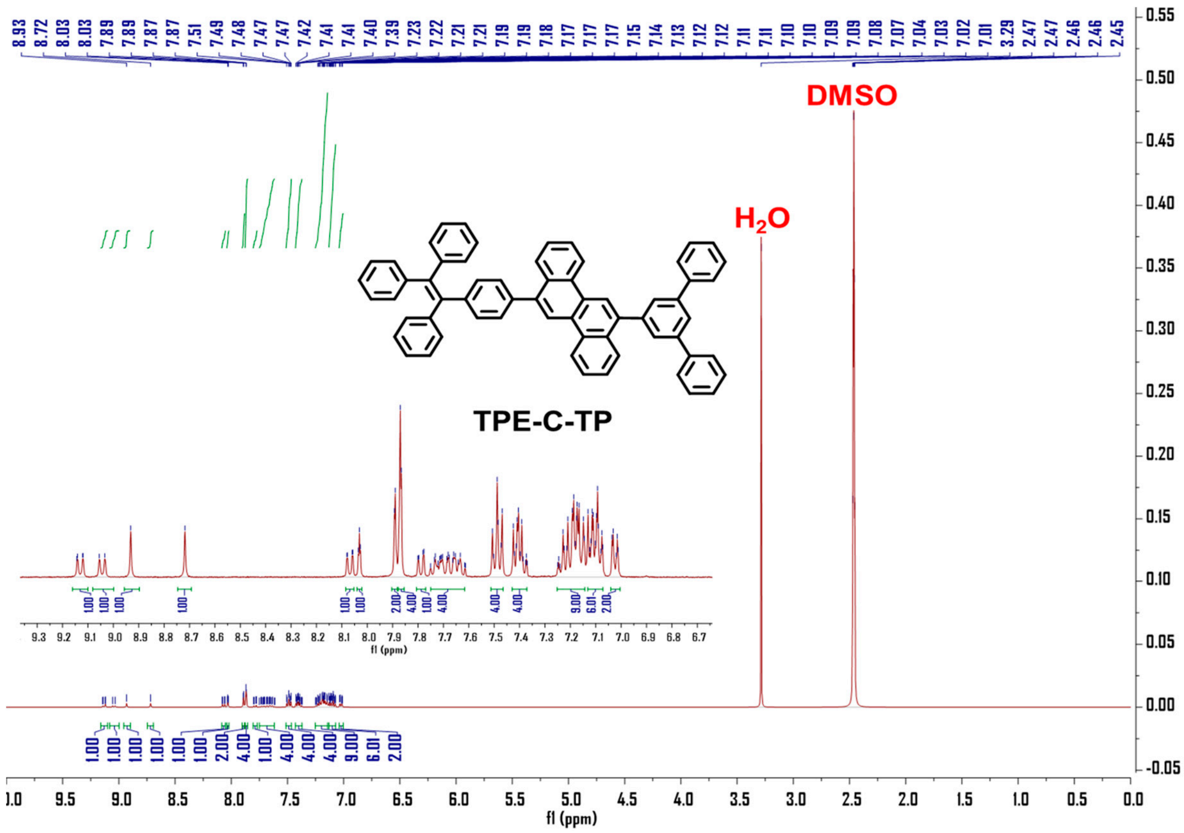

Figure S3. ^1H-NMR spectrum of TPE-C-TP.

Theoretical calculations

The characteristics of the natural transition orbitals (NTOs) for the S1 state of TPA-C-TP are depicted in Figure S4, with respect to the hole and particle. The electron density distribution in the hole shows a spread across both the TPA group and the chrysene group. In the particle, the electron density distribution extends from the chrysene group to the phenyl group attached to it. This suggests the possibility of TPA-C-TP undergoing a mixing of LE state induced by chrysene and CT state induced by TPA, indicative of HLCT properties. As a result, it is anticipated that not only will the PLQY of TPA-C-TP increase, but also by utilizing its CT characteristics, it will enable the generation of numerous singlet excitons, ultimately leading to a relatively high external quantum efficiency (EQE). These findings align with the solvatochromism results presented earlier. Density functional theory calculations were performed to confirm the change in properties caused by introducing side groups of TPA, TP, and TPE into the chrysene core, as shown in Figure S5. The electron densities of the highest occupied molecular orbitals (HOMOs), the lowest unoccupied molecular orbitals (LUMOs), and the band gap ($\Delta E_{H\text{-}L}$) of the two newly synthesized chrysene derivatives are summarized in Table 1. The electron density of TPA-C-TP was mainly distributed over the TPA side group in the HOMO energy level. In the case of the LUMO level, unlike the HOMO level, the electron density distribution was concentrated in the core, as well as slightly overlapped with TPA and expanded to TP. As TPA with an electron-donating effect was introduced, weak bipolar characteristics were observed in TPA-C-TP. This is consistent with the previous solvatochromism experimental results. In the case of TPE-C-TP, composed of polycyclic aromatic hydrocarbon, the HOMO and LUMO levels were distributed throughout the molecule, showing similar results. The measured HOMO levels of TPA-C-TP and TPE-C-TP were −5.61 and −5.85 eV, and the LUMO levels were calculated as −2.63 and −2.68 eV, respectively. The HOMO level of TPA-C-TP was 0.24 eV higher than that of TPE-C-TP, due to the electron-donating effect of TPA, an aromatic amine. The tendency between the measured and calculated values of the HOMO level and LUMO level were consistent. The calculated band gap values ($\Delta E_{H\text{-}L}$) of TPA-C-TP and TPE-C-TP were 3.53 eV and 3.72 eV, respectively. The measured band gap values of TPA-C-TP and TPE-C-TP were 2.98 eV and 3.17 eV, respectively. This showed the same trend as the calculated value. The band gap of TPA-C-TP is 0.19 eV smaller than that of TPE-C-TP. This reduction is interpreted as a result of the electron-donating effect of TPA, which increases the HOMO of TPA-C-TP and thus decreases the band gap. This interpretation aligns with the observation that TPA-C-TP exhibits photoluminescence at longer wavelengths in solution compared to TPE-C-TP.

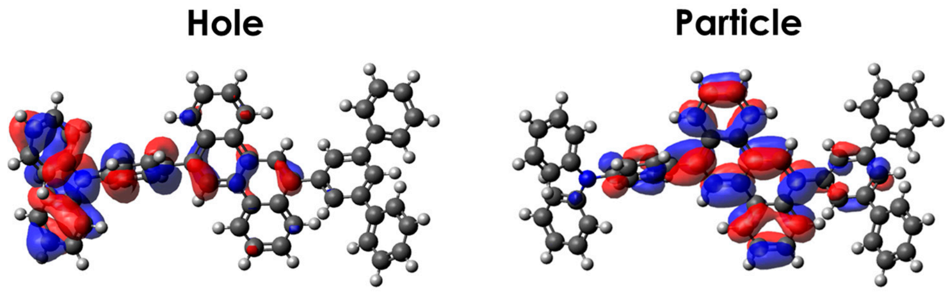

Figure S4. The characteristics of the natural transition orbitals (NTO) for the S1 state of TPA-C-TP, calculated using TDA-DFT B3LYP/6-31G(d) function.

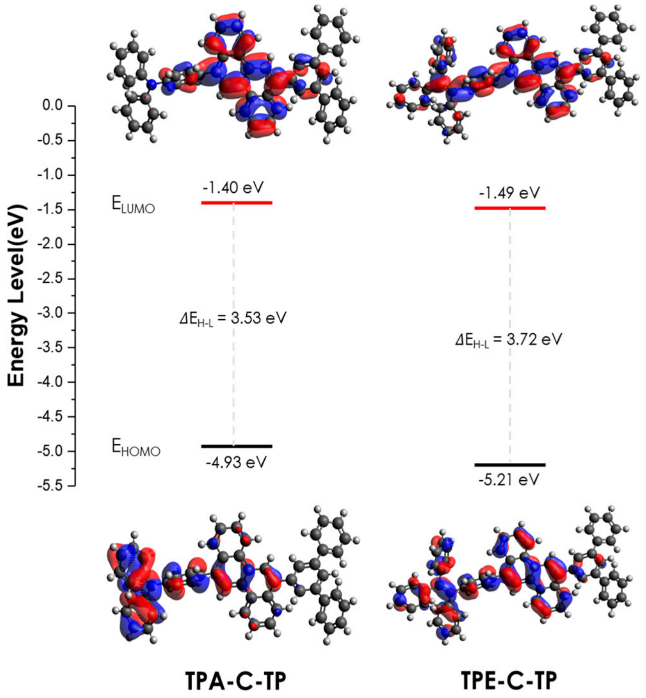

Figure S5. Frontier molecular orbital distributions of TPA-C-TP and TPE-C-TP calculated at the B3LYP/6-31G(d) level of theory.

Thermal properties

As shown in Figure S6 and Table 1, the glass transition temperature (T_g), melting temperature (T_m), and decomposition temperature (T_d) values of the synthesized materials were confirmed through differential scanning calorimetry and thermogravimetric analysis experiments. The T_d values corresponding to 5% weight loss of TPA-C-TP and TPE-C-TP were measured as 467 °C and 460 °C, respectively. T_g of TPA-C-TP was not observed, but its T_m was measured at 256 °C. On the other hand, TPE-C-TP had a T_g of 150 °C and a T_m of 297 °C. The high thermal stability of these materials helps to ensure the stability of OLED devices, even under the heat generated during operation [37, 38].

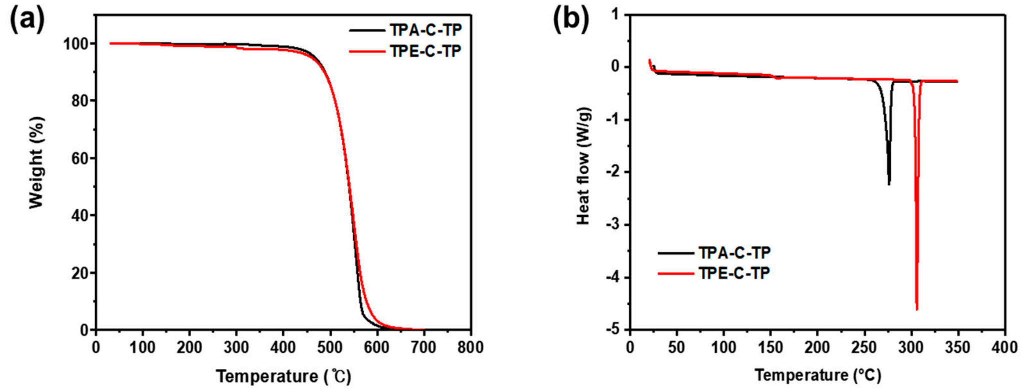

Figure S6. (a) Thermogravimetric analysis curves and (b) differential scanning calorimetry curves of TPA-C-TP and TPE-C-TP.

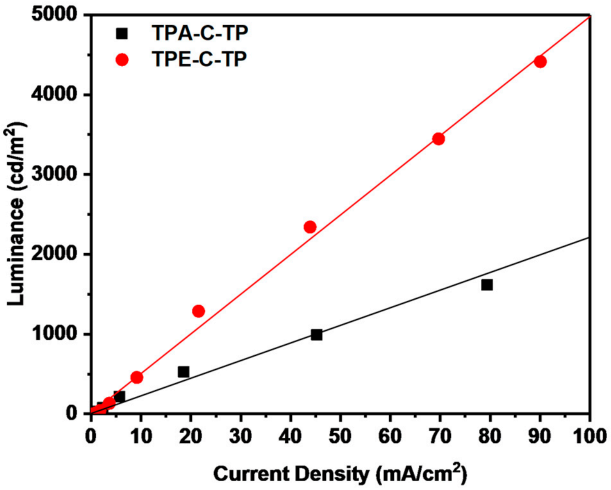

Figure S7. EL characteristic of luminance-current density curves for devices using TPA-C-TP and TPE-C-TP as the emitter.

Reference

37. Lin, S.-J.; Cheng, Y.-C.; Chen, C.-H.; Zhang, Y.-Y.; Lee, J.-H.; Leung, M.-k.; Lin, B.-Y.; Chiu, T.-L., New high-Tg bipolar benzimidazole derivatives in improving the stability of high-efficiency OLEDs. *J. Mater. Chem. C* **2023,** 11, (1), 161-171.

38. Chen, B.; Liao, C.; Li, D.; Liu, H.; Wang, S., Ultra-deep-blue thermally actrivated delayed fluorescence emitters constructed by carbazole derivatives enable efficient solution-processed OLED with a CIEy of < 0.05. *J. Mater. Chem. C* **2023,** 11, (26), 8767-8775.
